# Effects of T3 Administration on *Ex Vivo* Rat Hearts Subjected to Normothermic Perfusion: Therapeutic Implications in Donor Heart Preservation and Repair

**DOI:** 10.3389/ti.2023.10742

**Published:** 2023-02-07

**Authors:** Iordanis Mourouzis, Dimitris Kounatidis, Vassiliki Brozou, Dimitris Anagnostopoulos, Athanasia Katsaouni, Athanasios Lourbopoulos, Constantinos Pantos

**Affiliations:** Department of Pharmacology, Medical School, National and Kapodistrian University of Athens, Athens, Greece

**Keywords:** heart, normothermic perfusion, thyroid hormone, cold cardioplegia, kinase signalling

## Abstract

The present study investigated the effects of triiodothyronine (T3) administration in *ex vivo* model of rat heart normothermic perfusion. T3 is cardioprotective and has the potential to repair the injured myocardium. Isolated hearts were subjected to normothermic perfusion (NP) with Krebs-Henseleit for 4 h with vehicle (NP) or 60 nM T3 in the perfusate (NP + T3). Left ventricular end diastolic pressure (LVEDP), left ventricular developed pressure (LVDP), perfusion pressure (PP) and percentage of change of these parameters from the baseline values were measured. Activation of stress induced kinase signaling was assessed in tissue samples. Baseline parameters were similar between groups. LVEDP was increased from the baseline by 13% (70) for NP + T3 vs. 139% (160) for NP group, *p* = 0.048. LVDP was reduced by 18.2% (5) for NP + T3 vs. 25.3% (19) for NP group, *p* = 0.01. PP was increased by 41% (19) for NP + T3 vs.91% (56) for NP group, *p* = 0.024. T3 increased activation of pro-survival Akt by 1.85 fold (*p* = 0.047) and AMPK by 2.25 fold (*p* = 0.01) and reduced activation of pro-apoptotic p38 MAPK by 3fold (*p* = 0.04) and p54 JNK by 4.0 fold (*p* = 0.04). Administration of T3 in normothermic perfusion had favorable effects on cardiac function and perfusion pressure and switched death to pro-survival kinase signaling.

## Graphical Abstract

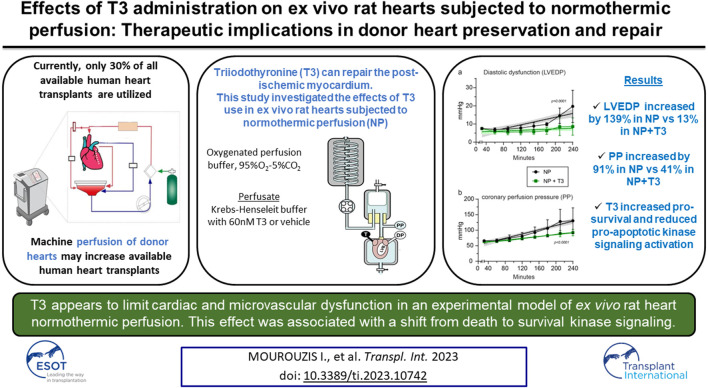

## Introduction

Heart transplantation remains the cornerstone therapy for end stage heart failure with an overall median survival of 12.5 years (1). Preservation solutions and techniques are crucial for donor organ quality which affects morbidity and survival after transplantation (2). Currently, cold storage is the standard method for organ preservation. However, prolonged cold storage may increase the risk of early graft dysfunction due to residual ischemia, reperfusion and rewarming injury (3). Furthermore, the demand for the use of marginal donor organs requires methods for organ assessment and repair. Machine normothermic or hypothermic perfusion has been recently attempted as a promising preservation technique (4). Machine perfusion may enable the use of cardioprotective agents to enhance tolerance of the donor heart to ischemia and prevent cardiac remodeling (5, 6). Thus, this new therapeutic challenge may increase both donor pool and post transplantation survival (7).

Thyroid hormone has traditionally been used in heart transplantation, but its mode of action is not fully understood (8–11). It is now recognized that thyroid hormone, beyond its classical actions on metabolism, has cardioprotective and reparative actions due to its differential effects on healthy and injured myocardium. Thus, thyroid hormone pretreatment can precondition the heart against ischemia-reperfusion (12) and triiodothyronine (T3) administration at reperfusion can limit reperfusion injury and cardiac remodeling (13, 14). T3 action is mediated by a delicate balance between pro-apoptotic and pro-survival kinase signaling pathways (15). This kinase signaling balance seems to be critical for cardiac injury and remodeling after an ischemic insult (16). More recently, the cardioprotective and reparative effects of T3 have also been demonstrated in humans (17, 18).

Based on this evidence, the aim of the present study was to compare normothermic perfusion to cold standard cardioplegia technique and investigate the potential of T3 administration to optimize normothermic perfusion in an *ex vivo* rat heart experimental model. This issue is of therapeutic importance and has not previously been studied.

## Materials and Methods

### Animals

Wistar male rats, 380–500 g, were used for this study. The rats were handed in accordance with the Guide for the Care and Use of Laboratory Animals published by the US National Institutes of Health (NIH Pub. No. 8323, Revised 1996). The protocol of the study was approved by the Animal Care and Use Committee of Department of Pharmacology, Medical School, National and Kapodistrian University of Athens (license 842/20-02-2017, ΕL 25BIOexp 10).

### Experimental Protocol

**FIGURE 1:**
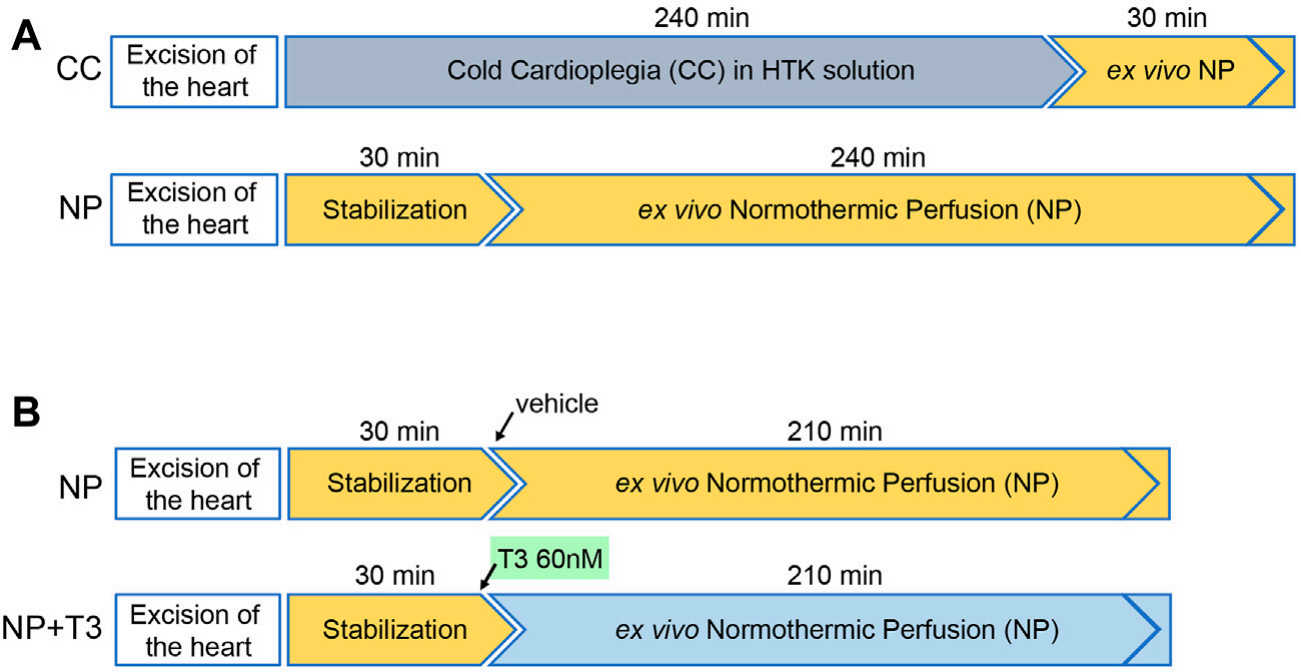
Schematic diagram showing the experimental design of the study. **(A)** Experiments performed to compare Cold Cardioplegia (CC) to normothermic perfusion (NP), **(B)** Experiments performed to investigate the effects of triiodothyronine (T3) in rat hearts with NP.

In order to assess the effects of cold cardioplegic arrest versus normothermic perfusion on cardiac function and perfusion pressure, the following experiments were performed (Figure 1A).

a. Hearts excised and subjected to cold cardioplegic (CC) arrest for 240 min and then perfused in Langendorff apparatus allowing 30 min recovery (group CC, *n* = 10),
b. Hearts excised and subjected to normothermic perfusion (NP) in Langendorff apparatus for 210 min after an initial period of 30 min perfusion (stabilization period), (group NP, *n* = 10).

In order to assess the effects of T3 administration on cardiac function and perfusion pressure during *ex vivo* normothermic perfusion, the following experiments were performed (Figure 1B).

a. Hearts excised and subjected to normothermic perfusion in Langendorff apparatus for 210 min after an initial period of 30 min perfusion (stabilization period). At the end of stabilization, KH buffer was supplemented with vehicle (group NP, *n* = 10),
b. Hearts excised and subjected to normothermic perfusion in Langendorff apparatus for 210 min after an initial period of 30 min perfusion (stabilization period). At the end of stabilization, KH buffer was supplemented with 60nM T3 (group NP + T3, *n* = 8),

During stabilization both groups were treated the same. At the end of the perfusion, LV was isolated, frozen in liquid nitrogen and kept at −80°C for molecular analysis.

### Anesthesia and Recovery After Cold Static Storage

Rats were anaesthetized with ketamine HCl and heparin 1000 IU was given intravenously. Following anesthesia, thoracotomy was performed to expose the heart and major vessels. The heart and large vessels were excised and immersed into ice cold histidine-tryptophan-ketoglutarate (HTK) cardioplegia solution identical to the solution used for cardiopreservation during heart transplantation (composition in mmol/L NaCl 15, KCl 9, MgCl 2 4, Adenosine 5, a-ketoglutarate 1, Tryptophan 2, Histidine 180, Histidine-HCl, 8, Mannitol 30). Changes in cold HTK solution were performed to achieve removal of blood (within 90 s) and then the heart was transferred to 100 mL of new cold HTK cardioplegia solution and stored at 4°C for 240 min. After this period, hearts were cannulated *via* the ascending aorta and perfused in the Langendorff apparatus with KH buffer allowing 30 min recovery. An intraventricular balloon was inserted into the left ventricle *via* the left atrium and allowed measurement of left ventricular pressure under isovolumic conditions. In order preload to be similar in both groups and equal to normal preload of the isolated rat heart, balloon volume was set at 250 μL. Constant flow was adjusted at 15 mL/min. The perfusion apparatus was heated to ensure a temperature of 37°C throughout the course of the experiment. Hearts were paced at 320 beats/min with a Harvard pacemaker. Pacing started 5–7 min after perfusion was achieved.

### Anesthesia and Recovery After Normothermic Perfusion

Rats were anaesthetized with ketamine HCl and heparin 1000 IU was given intravenously. The hearts were rapidly excised, placed in ice-cold Krebs-Henseleit (KH) buffer (composition in mmol/L: sodium chloride 118, potassium chloride 4.7, potassium phosphate monobasic 1.2, magnesium sulfate 1.2, calcium chloride 1.4, sodium bicarbonate 25, and glucose 11) and mounted on the aortic cannula of the Langendorff perfusion system. Perfusion with oxygenated (95% O_2_/5% CO_2_) Krebs-Henseleit buffer was established within 60 s after thoracotomy. The heart perfused to a non-working isolated rat heart preparation at a constant flow according to the Langendorff technique as previously described (12, 14, 19, 20). A water filled balloon, connected to a pressure transducer, was advanced into the left ventricle through an incision in the left atrium. Pressure signal was transferred to a computer using a data analysis software (IOX, Emka Technologies) which allowed continuous monitoring and recording (21). The intraventricular balloon allowed measurement of left ventricular pressure under isovolumic conditions. Left ventricular balloon volume was adjusted to produce an average initial left ventricular end-diastolic pressure of 6–8 mmHg in all groups and was held constant thereafter throughout the experiment. Since the balloon was not compressible, left ventricular contraction was isovolumic. As intraventricular volume was maintained at a constant value, preload, did not change. Thus, the left ventricular peak systolic pressure and the left ventricular developed pressure (LVDP), defined as the difference between left ventricular peak systolic pressure and left ventricular end-diastolic pressure, represented indexes of systolic function obtained under isometric conditions.

The perfusion apparatus was heated to ensure a temperature of 37°C throughout the course of the experiment. Constant flow was adjusted at 15 mL/min. Hearts were paced at 320 beats/min with a Harvard pacemaker. Pacing started 5–7 min after perfusion was achieved.

### Administration of Triiodothyronine

3,5,3′-triiodothyronine (T3) was purchased from Sigma Chemicals (St Louis MO, USA). T3 was dissolved in ethanol by adding a small volume of 25% NaOH and diluted in 0.9% normal saline to obtain a stock solution. T3 concentration in stock solution was 1 mg/mL. Stock solutions were kept at −20°C and before each experiment a quantity of this solution containing T3 was added in Krebs-Henseleit (KH) buffer to a final concentration of 60 nM. This dose has previously been shown to be cardioprotective against ischemia-reperfusion injury in an isolated rat heart experimental model (14). T3 administration initiated after the first 30 min of perfusion (stabilization period) in the isolated heart apparatus. Vehicle (T3 diluent) was injected in hearts subjected to normothermic perfusion without T3.

### Measurement of Mechanical Function

Left ventricular function was assessed by recording the left ventricular developed pressure (LVDP, mmHg) and the positive and negative first derivative of LVDP (+dp/dt and –dp/dt). Diastolic function was assessed by monitoring isovolumic left ventricular end-diastolic pressure (LVEDP) as a measure of diastolic chamber distensibility. Perfusion pressure under constant flow conditions was used to assess coronary vessel resistance (PP, mmHg). All parameters were also expressed as percentage of change from the baseline values.

### Molecular Analysis

In order to investigate potential molecular mechanisms underlying the effects of T3 in normothermic perfusion, the pattern of stress induced kinase signaling activation was assessed in NP and NP + T3 hearts at the end of perfusion. Molecular analysis was performed as previously described (21,22). A sample from the left ventricular tissue (200–220 mg) was homogenized in ice-cold buffer containing 10 mM Hepes (pH: 7.8), 10 mM KCl, 0.1 mM EDTA, 0.1 mM EGTA, 0.5 mM PMSF, 1 mM DTT and 10 μg/mL leupeptin. 200 μL of 10% Igepal was added and samples were left in ice for 30 min. Homogenization was repeated and the homogenate was centrifuged at 1000 g for 5 min, 4°C. The supernatant representing the cytosol-membrane fraction was kept at −80°C for further processing. Protein concentrations were determined by the bicinchoninic acid (BCA) method, using bovine serum albumin as a standard. Samples were prepared for sodium dodecyl sulfate polyacrylamide gel electrophoresis (SDS-PAGE) by boiling for 5 min in Laemmli sample buffer containing 5% 2-mercaptoethanol. Aliquots (40 μg) were loaded onto 9% (w/v) acrylamide gels and subjected to SDS-PAGE in a Bio-Rad Mini Protean gel apparatus. Following SDS-PAGE, proteins were transferred to a nitrocellulose membrane (Hybond ECL) at 100 V and 4°C, for 1.5 h using Towbin buffer for Western blotting analysis. Subsequently, filters were probed with specific antibodies against total p38 MAPK and dual phospho-p38 MAPK, total c-jun NH2-terminal kinases (JNKs) and dual phospho-JNKs, total Akt and dual phospho-Akt (Cell Signaling Technology, dilution 1:1000), total AMPK and phospho (Thr172)-AMPK (Cell Signaling Technology, dilution 1:1000) overnight at 4°C. Filters were incubated with appropriate anti-mouse (Amersham) or anti-rabbit (Cell Signalling) HRP secondary antibodies and immunoreactivity was detected by enhanced chemiluminescence using Lumiglo reagents (New England Biolabs). Chemiluminescence was detected by the image analysis system FluorChem HD2 (AlphaInnotech Corporation, 14743, Catalina Street, San Leandro, CA) equipped with a CCD camera and analysis software. Five samples from each group were loaded on the same gel for comparisons between groups. Data were expressed as the ratio of phosphorylated to total protein expression.

### Statistics

Values are presented as mean (Standard Deviation). Normal distribution of variables was estimated with Shapiro-Wilk test of normality. Normally distributed data were compared using an independent t-test. Skewed data were analysed non-parametrically using Mann-Whitney U test. Serial measurements of LVEDP and PP were compared by mixed, repeated measures analysis of variance (mixed ANOVA) to test for the effect of treatment, time and the interaction (tests for “within-subjects” factor and “between-subjects” factor); the respective non-linear fit-curves with the 95% CI error were produced with non-linear fit analysis. When significant, differences within and between each group were tested by a *post hoc* analysis using the Bonferroni correction for multiple comparisons if needed. A two-tailed test with a *p* value less than 0.05 was considered significant. Analysis was performed using the GraphPad 8 software.

## Results

### Comparison of Normothermic Perfusion to Cold Cardioplegic Arrest

**FIGURE 2:**
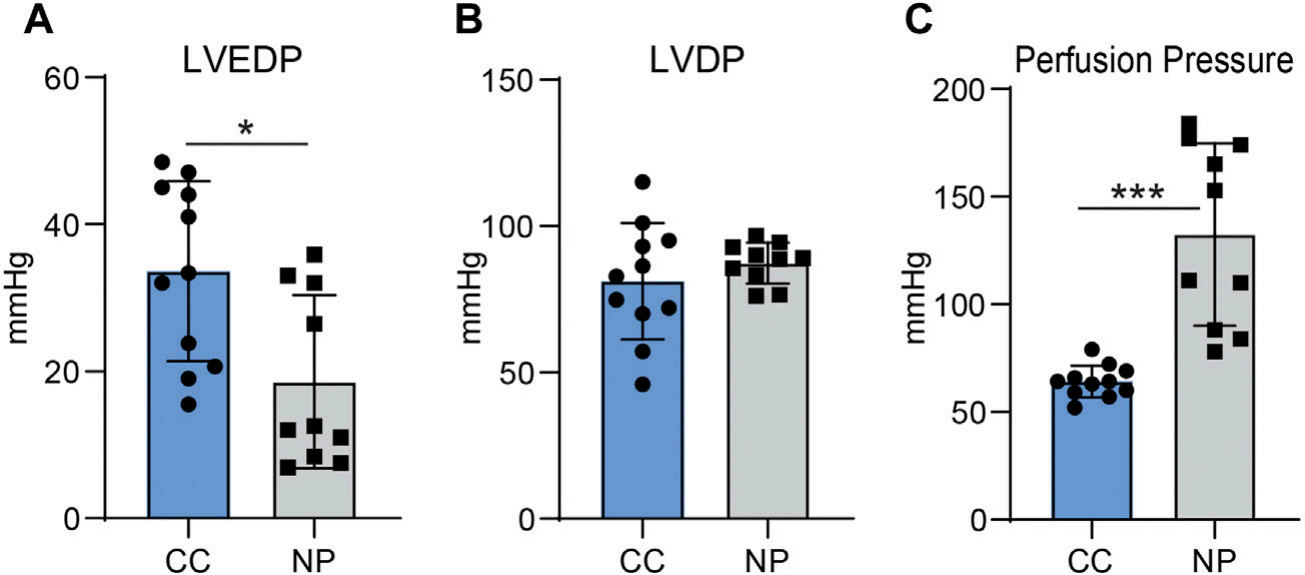
Left ventricular end-diastolic pressure (LVEDP, **(A)**, left ventricular developed pressure [LVDP, **(B)**] and perfusion pressure [PP, **(C)**] in hearts preserved with cold cardiop legia (CC, *n* = 10) and hearts with normothermic perfusion (NP, *n* = 10). Values represent Means, bars stand for standard deviation. *p < 0.05, ***p; 0.001.

Cardiac function and perfusion pressure were assessed at the end of the recovery period in CC group and the end of normothermic perfusion in NP group. After cold cardioplegia, LVEDP was 33.6 (11.7) mmHg in CC group vs.18.5 (13.3) in NP group, *p* = 0.02. LVDP was similar between the groups; 81 (18.9) mmHg for CC and 87.3 (7.0) for NP, *p* = n.s. Perfusion Pressure was 64.2 (7.0) mmHg in CC group vs. 132 (51) mmHg in NP group, *p* = 0.001 (Figure 2).

### Effects of T3 Administration in Normothermic Perfusion

**FIGURE 3:**
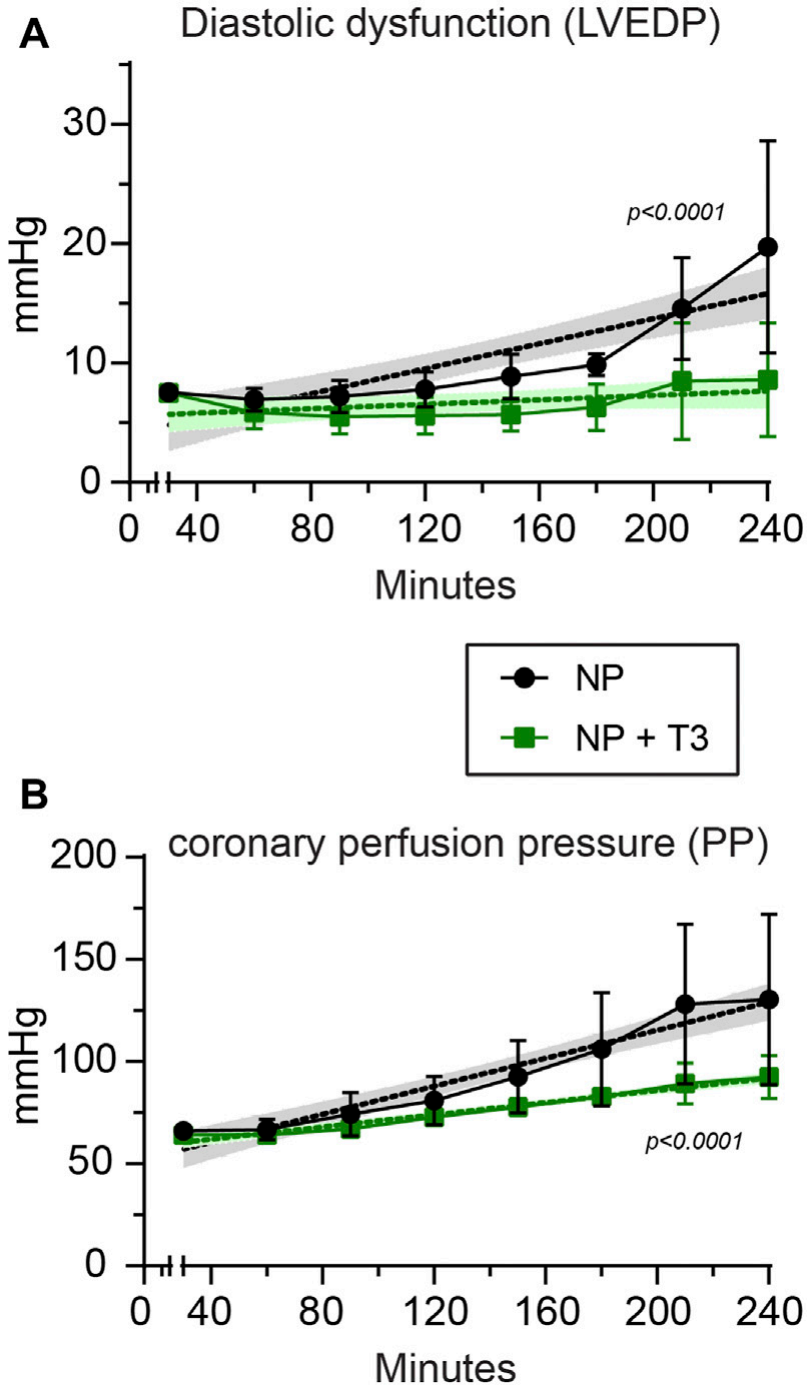
Left ventricular end-diastolic pressure [LVEDP, **(A)**], and perfusion pressure [PP, **(B)**] in hearts subjected to normothermic perfusion in Langendorff apparatus for 210 min after an initial period of 30 min perfusion (stabilization period). At the end of stabilization, KH buffer was supplemented either with vehicle (NP, *n* = 10) or with 60 nM T3 (NP + T3, *n* = 8). Values represent Means, bars stand for standard deviation. The respective non-linear fit-curves with the 95% Confidence Intervals produced with non-linear fit analysis are shown with dotted lines.

Cardiac function and perfusion pressure were measured at the end of stabilization period (baseline parameters) and the end of normothermic perfusion. Baseline parameters were similar between NP and NP + T3 groups (Table 1). LVEDP was 19.7 (12) mmHg in NP group and 8.6 (5.8) mmHg in T3 treated group *p* = 0.026 (Figure 3A). LVDP was 87 (20) mmHg in NP group and 90 (21) mmHg in T3 treated group, *p* = 0.49. PP was 130 (39.1) mmHg in NP and 92 (10.6) mmHg in T3 treated group, *p* = 0.019 (Table 1).

**TABLE 1:** Cardiac function and perfusion pressure at the end of stabilization (baseline parameters) and the end of normothermic perfusion. Data are presented as Mean (SD).

	NP (*n* = 10)			NP + T3 (*n* = 8)		
	End of stabilization	End of perfusion	% Change from baseline	End of stabilization	End of perfusion	% Change from baseline
LVDP (mmHg)	116.7 (9.0)	87.3 (6.9)	−25 (5)%	112.3 (8.2)	90.0 (7.3)	−18 (5)%*
LVEDP (mmHg)	7.6 (0.42)	19.7 (12)	139 (160)%	7.5 (0.43)	8.5 (5.6)*	13 (70)%*
+dp/dt (mmHg/sec)	3740 (826)	3038 (612)	−18 (10)%	3593 (803)	3032 (850)	−15 (6)%
−dp/dt (mmHg/sec)	2189 (268)	1490 (216)	−32 (6)%	2045 (227)	1515 (268)	−25 (9)%^#^
PP (mmHg)	67 (4.4)	130 (41)	91 (56)%	66 (2.4)	92 (10)*	41 (19)%*

LVDP, left ventricular developed pressure; LVEDP, left ventricular end-diastolic pressure; +dp/dt, rate of increase of LVDP; −dp/dt, rate of decrease of LVDP; PP, perfusion pressure.

*p < 0.05 vs. NP; ^#^ *p* = 0.08 vs. NP.

Functional parameters and perfusion pressure were also expressed as percentage of change from the baseline values. LVEDP gradually increased by 139% from baseline in NP group, and only by 13% in NP + T3 group, *p* = 0.048 (Table 1). The decline in LVDP was found to be less after T3 treatment; 18.2% in NP + T3 vs. 25.3% in NP, *p* = 0.01 (Table 1). PP increased by 91% from baseline in NP group and by 41% in NP + T3 group, *p* = 0.024 (Table 1).

Measurements of cardiac function and perfusion pressure were also performed every 30 min from the end of stabilization period to the end of normothermic perfusion (Figure 3). Mixed Repeated Measures ANOVA analysis for LVEDP (dependent variable) at different time points between the groups showed that the main effect of treatment (T3, “between-subjects” factor) on LVEDP was statistically significant (F = 4.0, *p* = 0.04). Thus, T3 treatment induced a sustained improvement of LVEDP over time. Furthermore, there was a statistically significant effect (F = 9.1, *p* = 0.002) regarding the main effect of time (“within-subjects” factor). There was also a statistically significant interaction effect between time and treatment (*p* = 0.044).

Mixed Repeated Measures ANOVA analysis for PP (dependent variable) at different time points between the groups showed that the main effect of treatment (T3, “between-subjects” factor) on PP was statistically significant (F = 5.5, *p* = 0.03). Thus, T3 treatment induced a sustained improvement of PP over time. Furthermore, there was a statistically significant effect (F = 28.8, *p* = 0.000002) regarding the main effect of time (“within-subjects” factor) on deterioration of PP. Α statistically significant interaction effect between time and treatment was found (*p* = 0.024).

At the end of normothermic perfusion, the ratio of LV weight to body weight was 2.35 (0.3) for the NP hearts and 2.39 (0.26) for NP + T3 hearts, *p* = n.s.

### Effects of T3 Administration on the Activation of Stress Induced Kinases

Stress induced kinase signaling activation was assessed in NP and NP + T3 hearts at the end of normothermic perfusion. T3 administration during normothermic perfusion resulted in suppression of proapoptotic signalling and upregulation of the pro-survival signalling pathways.

**FIGURE 4:**
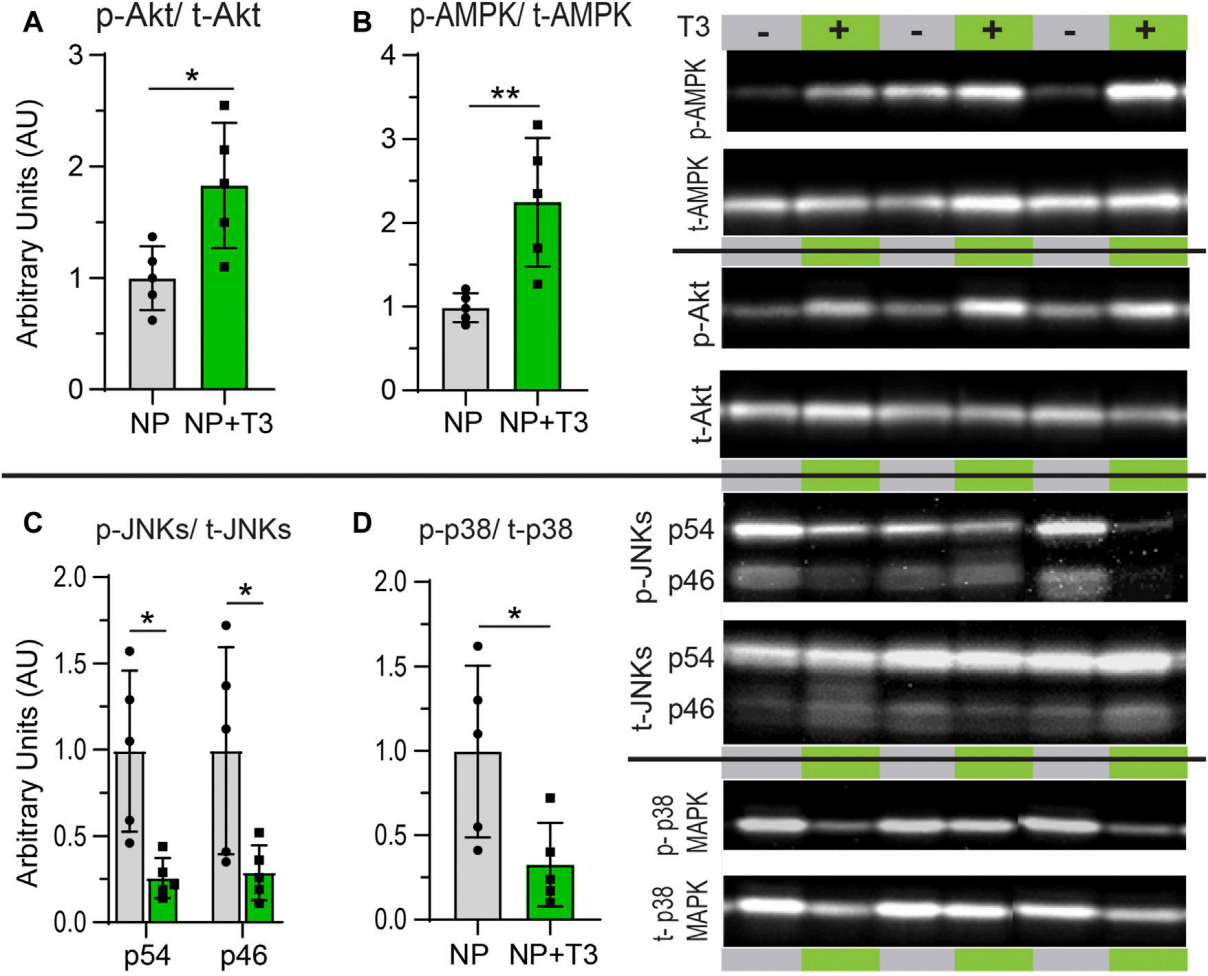
Representative western blots and densitometric assessment in arbitrary units of the ratio of phosphorylated Akt to total Akt expression **(A)**, phosphorylated AMPK to total AMPK expression **(B)**, phosphorylated p54 and p46 JNKs to total JNKs expression **(C)** and phosphorylated p38 MAPK to total p38 MAPK expression **(D)** in hearts at the end of normothermic perfusion supplemented either with vehicle (NP) or with 60 nM T3 (NP + T3). Data represent Means from *n* = 5 hearts per group. Bars stand for standard deviation. *p < 0.05, **p < 0.01.

Phosphorylated to total Akt and phosphorylated to total AMPK levels were both significantly increased by 1.85 (*p* = 0.047) and 2.25-fold (*p* = 0.01) in T3 treated hearts (NP + T3) as compared to NP hearts, *p* < 0.05 (Figures 4A,B).

Phosphorylated to total p38 MAPK levels were lower by 3 fold in NP + T3 as compared to NP hearts, *p* = 0.04. Phosphorylated to total p54 and p46 JNKs levels were lower by 4.0 (*p* = 0.04) and 3.7-fold (*p* = 0.03) respectively in NP + T3 as compared to NP hearts (Figures 4C,D).

## Discussion

Normothermic machine perfusion is an emerging preservation modality which maintains organ metabolism and thus, prevents the harmful effects of cold ischemia. Machine perfusion is non inferior to cold cardioplegia and is particularly beneficial for high risk donors and donation of hearts after circulatory death (23). However, optimal microcirculatory support is crucial for successful normothermic perfusion. After removal of the organ, the disconnection from all autonomic and endocrine systems may cause deregulation of vascular tone which leads to impairment in microcirculation and tissue hypoxia which in turn results in tissue injury, edema and remodeling (viscous circle). Several pharmacological interventions aiming to induce vasodilation, reduce edema and enhance metabolism have been investigated as potential donor organ preservation modalities (24). Triiodothyronine has long been used for hemodynamic support and as replacement therapy along with other hormones for donor organ preservation and resuscitation (8–11). Beyond these classical actions, T3 at doses several folds above normal is shown to reduce myocardial ischemia-reperfusion injury, prevent cardiac remodeling and promote regeneration (12, 14, 25). More importantly, this cardioprotective and reparative action has recently been shown in humans (17). T3 regulates vascular function, induces angiogenesis and mitochondrial biogenesis (26–28). Based on this evidence, we hypothesized that high dose T3 could potentially optimize cardiac preservation in the setting of normothermic perfusion.

In the present study, an *ex vivo* rat heart normothermic perfusion model was established to identify differences between normothermic perfusion and cold cardioplegia and to investigate the potential of high dose T3 to optimize normothermic perfusion. A decline of 5%–10% of left ventricular developed pressure per hour perfusion has been reported in this experimental model (29). In this setting, coronary perfusion pressure (an indirect index of microcirculation) was found to be higher in hearts subjected to normothermic perfusion compared to cold cardioplegia, probably indicating impaired microcirculation after normothermic perfusion. It is of note that microcirculation is impaired after reperfusion in by-pass surgery (30), acute myocardial infarction (31) and heart transplantation (32, 33) despite vascular blood flow restoration in all these conditions. Furthermore, the presence of microvascular dysfunction has been associated with postischemic cardiac remodeling and impaired healing (31, 34). The optimization of microcirculation after reperfusion remains a therapeutic challenge. In this context, according to our data, T3 administration in normothermic perfusion resulted in significant lower coronary perfusion pressure as compared to untreated hearts. Furthermore, the decline in LVDP was also significantly less in treated hearts and LVEDP was increased by only 13% of the initial value after normothermic perfusion in treated hearts as compared to 139% increase in the untreated group. Heart weight at the end of perfusion was similar between the groups probably indicating that T3 had no effect on tissue edema. Collectively, these data suggest that T3 may have favorable effects on microcirculation and prevent tissue hypoxia induced functional changes after normothermic perfusion. These observations are consistent with previous reports showing that T3 treatment improves tissue hypoxia in experimental settings of impaired microcirculation such as ischemia–reperfusion and sepsis. Thus, T3 administration early at reperfusion improved post-ischemic recovery of function and limited apoptosis particularly in the mid layer of the myocardium in which extensive microvascular network exists (14). Furthermore, T3 administration prevented cardiac and liver tissue hypoxia and microvascular dysfunction in experimental sepsis (35). Interestingly, the potential of T3 to prevent tissue hypoxia has also been demonstrated in patients with sepsis in a preliminary study (36).

The mechanisms underlying the cardioprotective effect of T3 have been under intense investigation. Thyroid hormone is shown to protect the heart against ischemic/reperfusion injury *via* regulation of intracellular kinase signaling pathways (15). Cellular kinase signaling pathways play an important pathophysiologic role in the ischemic injury, post-ischemic cardiac remodeling and heart failure (16). It is now recognized that a critical balance between pro-apoptotic and pro-survival pathways determines myocardial injury or survival after ischemia. In this context, activation of p38 MAPK results in apoptosis, vascular permeability, depression of cardiac function and immune cell activation and cytokine upregulation (37). Accordingly, inhibition of p38 MAPK activation lowered systematic levels of pro-inflammatory cytokines in a brain dead donor model (38). Furthermore, addition of a p38 MAPK inhibitor to the Euro Collins and University of Wisconsin solutions mitigated ischemia/reperfusion injury in lung, liver and kidney grafts (39). p38 MAPK is critical for organ repair after stress and inhibition of p38 MAPK enables proliferation of adult mammalian cardiomyocytes and heart regeneration (40). JNK activation also leads to apoptosis and inhibition of this kinase reduced histological rejection and improved graft survival in a rat model of transplantation (41).

Activation of pro-survival Akt signaling pathway prevents apoptosis, induces angiogenesis and regulates calcium handling (42). Interestingly, crosstalk between Akt and p38 MAPK signaling exists and seems to regulate the levels of cryoprotection vs. apoptosis in endothelial cells (43). Upon stress, AMPK appears to serve a critical role in regulating cardiac metabolism. Activation of AMPK improved left ventricular function and survival in experimental myocardial infarction (44).

On the basis of these data, the present study investigated potential changes in the ratio of phosphorylated to total kinase protein expression (an index of kinase activation) after T3 administration in normothermic perfusion. T3 significantly reduced the activation of the pro-apoptotic p38 MAPK and JNK signaling while enhanced pro-survival Akt signaling and AMPK. This molecular signature indicates that T3 administration can switch intracellular death signaling to survival with potential implications in postischemic remodeling and late heart failure.

### Clinical Implications -Limitations of the Study

*Ex vivo* perfusion is a new strategy for organ preservation and reconditioning prior to transplantation (4, 45). Extensive research in this field is underway to identify optimal perfusate composition and duration. The perfusate commonly used in clinical practice is cell based consisted of leukocyte depleted, packed red blood cells supplemented with anticoagulants and vasodilators. However, the use of red cells may not be suitable for prolonged periods of perfusion. There is now evidence that non cell solutions as Ringer’s lactate or Steen solution supplemented with nutrient and metabolic substrates may optimize normothermic perfusion (24). Along this line, the present study provides evidence that addition of high dose of T3 in K-H can preserve *ex vivo* rat hearts subjected to normothermic perfusion. Furthermore, for the first time, this therapeutic modality is shown to induce the activation of intracellular repair signaling. Until now, thyroid hormone has been added to solutions at replacement doses and along with other hormones to resuscitate and preserve donor organs (11). Thus, the cardioprotective effect of this hormone has not been evaluated.

There are limitations of this study which have to be taken into consideration. The study was performed in small animals and its findings are needed to be validated in large animals and humans. However, the rat species is shown to be of high translational validity for pharmacological studies of ischemia/reperfusion (17). The potential effects of T3 in extended periods of normothermic perfusion have not been investigated. Cardiac damage was assessed by functional indices and not histological examination. The benefit of T3 in cell-based solutions has not been investigated. However, it has recently been shown that T3 may have favorable effects on erythrocyte aggregation and hemorheology.

In conclusion, T3 appears to limit cardiac and microvascular dysfunction in an experimental model of *ex vivo* rat heart normothermic perfusion. This effect was associated with a switch of death to survival kinase signaling.

## Data Availability

The raw data supporting the conclusion of this article will be made available by the authors, without undue reservation.

## References

[1] Khush KK Cherikh WS Chambers DC Harhay MO Hayes D Jr. Hsich E The International Thoracic Organ Transplant Registry of the International Society for Heart and Lung Transplantation: Thirty-Sixth Adult Heart Transplantation Report - 2019; Focus Theme: Donor and Recipient Size Match. J Heart Lung Transpl (2019) 38(10):1056–66. 10.1016/j.healun.2019.08.004 PMC6816343 31548031

[2] Lepoittevin M Giraud S Kerforne T Barrou B Badet L Bucur P Preservation of Organs to Be Transplanted: An Essential Step in the Transplant Process. Int J Mol Sci (2022) 23(9):4989. 10.3390/ijms23094989 35563381 PMC9104613

[3] Minor T von Horn C. Rewarming Injury after Cold Preservation. Int J Mol Sci (2019) 20(9):2059. 10.3390/ijms20092059 31027332 PMC6539208

[4] Messer S Ardehali A Tsui S. Normothermic Donor Heart Perfusion: Current Clinical Experience and the Future. Transpl Int (2015) 28(6):634–42. 10.1111/tri.12361 24853906

[5] Minasian SM Galagudza MM Dmitriev YV Karpov AA Vlasov TD. Preservation of the Donor Heart: from Basic Science to Clinical Studies. Interact Cardiovasc Thorac Surg (2015) 20(4):510–9. 10.1093/icvts/ivu432 25538253

[6] Saeb-Parsy K Martin JL Summers DM Watson CJE Krieg T Murphy MP. Mitochondria as Therapeutic Targets in Transplantation. Trends Mol Med (2021) 27(2):185–98. 10.1016/j.molmed.2020.08.001 32952044

[7] Crespo-Leiro MG Costanzo MR Gustafsson F Khush KK Macdonald PS Potena L Heart Transplantation: Focus on Donor Recovery Strategies, Left Ventricular Assist Devices, and Novel Therapies. Eur Heart J (2022) 43(23):2237–46. 10.1093/eurheartj/ehac204 35441654

[8] Holndonner-Kirst E Nagy A Czobor NR Fazekas L Dohan O Kertai MD The Impact of L-Thyroxine Treatment of Donors and Recipients on Postoperative Outcomes after Heart Transplantation. J Cardiothorac Vasc Anesth (2019) 33(6):1629–35. 10.1053/j.jvca.2018.10.024 30467031

[9] Jeevanandam V. Triiodothyronine: Spectrum of Use in Heart Transplantation. Thyroid (1997) 7(1):139–45. 10.1089/thy.1997.7.139 9086582

[10] Novitzky D Mi Z Sun Q Collins JF Cooper DK. Thyroid Hormone Therapy in the Management of 63,593 Brain-Dead Organ Donors: a Retrospective Analysis. Transplantation (2014) 98(10):1119–27. 10.1097/TP.0000000000000187 25405914

[11] Steen S Sjoberg T Liao Q Bozovic G Wohlfart B. Pharmacological Normalization of Circulation after Acute Brain Death. Acta Anaesthesiol Scand (2012) 56(8):1006–12. 10.1111/j.1399-6576.2012.02721.x 22651688

[12] Pantos CI Malliopoulou VA Mourouzis IS Karamanoli EP Paizis IA Steimberg N Long-term Thyroxine Administration Protects the Heart in a Pattern Similar to Ischemic Preconditioning. Thyroid (2002) 12(4):325–9. 10.1089/10507250252949469 12034058

[13] Pantos C Mourouzis I Markakis K Dimopoulos A Xinaris C Kokkinos AD Thyroid Hormone Attenuates Cardiac Remodeling and Improves Hemodynamics Early after Acute Myocardial Infarction in Rats. Eur J Cardiothorac Surg (2007) 32(2):333–9. 10.1016/j.ejcts.2007.05.004 17560116

[14] Pantos C Mourouzis I Saranteas T Clave G Ligeret H Noack-Fraissignes P Thyroid Hormone Improves Postischaemic Recovery of Function while Limiting Apoptosis: a New Therapeutic Approach to Support Hemodynamics in the Setting of Ischaemia-Reperfusion? Basic Res Cardiol (2009) 104(1):69–77. 10.1007/s00395-008-0758-4 19101750

[15] Pantos C Mourouzis I. Translating Thyroid Hormone Effects into Clinical Practice: the Relevance of Thyroid Hormone Receptor α1 in Cardiac Repair. Heart Fail Rev (2015) 20(3):273–82. 10.1007/s10741-014-9465-4 25501869

[16] Pantos C Mourouzis I Cokkinos DV. Myocardial Ischemia: Basic Concepts. In: DV Cokkinos C Pantos G Heusch H Taegtmeyer, editors. Myocardial Ischemia: From Mechanisms to Theurapeutic Potentials. New York: Springer (2006). p. 11–77.

[17] Pantos CI Trikas AG Pissimisis EG Grigoriou KP Stougiannos PN Dimopoulos AK Effects of Acute Triiodothyronine Treatment in Patients with Anterior Myocardial Infarction Undergoing Primary Angioplasty: Evidence from a Pilot Randomized Clinical Trial (ThyRepair Study). Thyroid (2022) 32(6):714–24. 10.1089/thy.2021.0596 35297659

[18] Ranasinghe AM Quinn DW Pagano D Edwards N Faroqui M Graham TR Glucose-insulin-potassium and Tri-iodothyronine Individually Improve Hemodynamic Performance and Are Associated with Reduced Troponin I Release after On-Pump Coronary Artery Bypass Grafting. Circulation (2006) 114:I245–50. 10.1161/CIRCULATIONAHA.105.000786 16820580

[19] Pantos C Mourouzis I Delbruyere M Malliopoulou V Tzeis S Cokkinos DD Effects of Dronedarone and Amiodarone on Plasma Thyroid Hormones and on the Basal and Postischemic Performance of the Isolated Rat Heart. Eur J Pharmacol (2002) 444(3):191–6. 10.1016/s0014-2999(02)01624-2 12063079

[20] Pantos C Mourouzis I Malliopoulou V Paizis I Tzeis S Moraitis P Dronedarone Administration Prevents Body Weight Gain and Increases Tolerance of the Heart to Ischemic Stress: a Possible Involvement of Thyroid Hormone Receptor Alpha1. Thyroid (2005) 15(1):16–23. 10.1089/thy.2005.15.16 15687816

[21] Pantos C Mourouzis I Markakis K Tsagoulis N Panagiotou M Cokkinos DV. Long-term Thyroid Hormone Administration Re-shapes Left Ventricular Chamber and Improves Cardiac Function after Myocardial Infarction in Rats. Basic Res Cardiol (2008) 103(4):308–18. 10.1007/s00395-008-0697-0 18274800

[22] Pantos C Malliopoulou V Paizis I Moraitis P Mourouzis I Tzeis S Thyroid Hormone and Cardioprotection: Study of P38 MAPK and JNKs during Ischaemia and at Reperfusion in Isolated Rat Heart. Mol Cel Biochem (2003) 242(1-2):173–80. 12619880

[23] Qin G Jernryd V Sjoberg T Steen S Nilsson J. Machine Perfusion for Human Heart Preservation: A Systematic Review. Transpl Int (2022) 35:10258. 10.3389/ti.2022.10258 35401041 PMC8983812

[24] Fard A Pearson R Lathan R Mark PB Clancy MJ. Perfusate Composition and Duration of *Ex-Vivo* Normothermic Perfusion in Kidney Transplantation: A Systematic Review. Transpl Int (2022) 35:10236. 10.3389/ti.2022.10236 35634582 PMC9130468

[25] Bogush N Tan L Naqvi E Calvert JW Graham RM Taylor WR Remuscularization with Triiodothyronine and β_1_-blocker Therapy Reverses post-ischemic Left Ventricular Dysfunction and Adverse Remodeling. Sci Rep (2022) 12(1):8852. 10.1038/s41598-022-12723-2 35614155 PMC9132945

[26] Makino A Wang H Scott BT Yuan JX Dillmann WH. Thyroid Hormone Receptor-Alpha and Vascular Function. Am J Physiol Cel Physiol (2012) 302(9):C1346–52. 10.1152/ajpcell.00292.2011 PMC3361953 22322976

[27] Bloise FF Santos AT de Brito J de Andrade CBV Oliveira TS de Souza AFP Sepsis Impairs Thyroid Hormone Signaling and Mitochondrial Function in the Mouse Diaphragm. Thyroid (2020) 30(7):1079–90. 10.1089/thy.2019.0124 32200709

[28] Chen J Ortmeier SB Savinova OV Nareddy VB Beyer AJ Wang D Thyroid Hormone Induces Sprouting Angiogenesis in Adult Heart of Hypothyroid Mice through the PDGF-Akt Pathway. J Cel Mol Med (2012) 16(11):2726–35. 10.1111/j.1582-4934.2012.01593.x PMC3448001 22681587

[29] Sutherland FJ Hearse DJ. The Isolated Blood and Perfusion Fluid Perfused Heart. Pharmacol Res (2000) 41(6):613–27. 10.1006/phrs.1999.0653 10816330

[30] Dekker NAM Veerhoek D Koning NJ van Leeuwen ALI Elbers PWG van den Brom CE Postoperative Microcirculatory Perfusion and Endothelial Glycocalyx Shedding Following Cardiac Surgery with Cardiopulmonary Bypass. Anaesthesia (2019) 74(5):609–18. 10.1111/anae.14577 30687934 PMC6590376

[31] Konijnenberg LSF Damman P Duncker DJ Kloner RA Nijveldt R van Geuns RM Pathophysiology and Diagnosis of Coronary Microvascular Dysfunction in ST-Elevation Myocardial Infarction. Cardiovasc Res (2020) 116(4):787–805. 10.1093/cvr/cvz301 31710673 PMC7061278

[32] Koch A Bingold TM Oberlander J Sack FU Otto HF Hagl S Capillary Endothelia and Cardiomyocytes Differ in Vulnerability to Ischemia/reperfusion during Clinical Heart Transplantation. Eur J Cardiothorac Surg (2001) 20(5):996–1001. 10.1016/s1010-7940(01)00905-8 11675187

[33] Lee JM Choi KH Choi JO Shin D Park Y Kim J Coronary Microcirculatory Dysfunction and Acute Cellular Rejection after Heart Transplantation. Circulation (2021) 144(18):1459–72. 10.1161/CIRCULATIONAHA.121.056158 34474597

[34] van Kranenburg M Magro M Thiele H de Waha S Eitel I Cochet A Prognostic Value of Microvascular Obstruction and Infarct Size, as Measured by CMR in STEMI Patients. JACC Cardiovasc Imaging (2014) 7(9):930–9. 10.1016/j.jcmg.2014.05.010 25212799

[35] Mourouzis IS Lourbopoulos AI Trikas AG Tseti IK Pantos CI. Triiodothyronine Prevents Tissue Hypoxia in Experimental Sepsis: Potential Therapeutic Implications. Intensive Care Med Exp (2021) 9(1):17. 10.1186/s40635-021-00382-y 33834320 PMC8032456

[36] Pantos C Apostolaki V Kokkinos L Trikas A Mourouzis I. Acute Triiodothyronine Treatment and Red Blood Cell Sedimentation Rate (ESR) in Critically Ill COVID-19 Patients: A Novel Association? Clin Hemorheol Microcirc (2021) 79(3):485–8. 10.3233/CH-211215 34151781

[37] Vassalli G Milano G Moccetti T. Role of Mitogen-Activated Protein Kinases in Myocardial Ischemia-Reperfusion Injury during Heart Transplantation. J Transpl (2012) 2012:928954. 10.1155/2012/928954 PMC3316985 22530110

[38] Oto T Calderone A Li Z Rosenfeldt FL Pepe S. p38 Mitogen-Activated Protein Kinase Inhibition Reduces Inflammatory Cytokines in a Brain-Dead Transplant Donor Animal Model. Heart Lung Circ (2009) 18(6):393–400. 10.1016/j.hlc.2009.05.706 19647484

[39] Koike N Takeyoshi I Ohki S Tokumine M Matsumoto K Morishita Y. Effects of Adding P38 Mitogen-Activated Protein-Kinase Inhibitor to Celsior Solution in Canine Heart Transplantation from Non-heart-beating Donors. Transplantation (2004) 77(2):286–92. 10.1097/01.TP.0000101039.12835.A4 14742995

[40] Engel FB Schebesta M Duong MT Lu G Ren S Madwed JB p38 MAP Kinase Inhibition Enables Proliferation of Adult Mammalian Cardiomyocytes. Genes Dev (2005) 19(10):1175–87. 10.1101/gad.1306705 15870258 PMC1132004

[41] Tabata A Morikawa M Miyajima M Bennett BL Satoh Y Huang J Suppression of Alloreactivity and Allograft Rejection by SP600125, a Small Molecule Inhibitor of C-Jun N-Terminal Kinase. Transplantation (2007) 83(10):1358–64. 10.1097/01.tp.0000264196.23944.90 17519787

[42] Shiojima I Schiekofer S Schneider JG Belisle K Sato K Andrassy M Short-term Akt Activation in Cardiac Muscle Cells Improves Contractile Function in Failing Hearts. Am J Pathol (2012) 181(6):1969–76. 10.1016/j.ajpath.2012.08.020 23031259 PMC3509766

[43] Gratton JP Morales-Ruiz M Kureishi Y Fulton D Walsh K Sessa WC. Akt Down-Regulation of P38 Signaling Provides a Novel Mechanism of Vascular Endothelial Growth Factor-Mediated Cytoprotection in Endothelial Cells. J Biol Chem (2001) 276(32):30359–65. 10.1074/jbc.M009698200 11387313

[44] Gundewar S Calvert JW Jha S Toedt-Pingel I Ji SY Nunez D Activation of AMP-Activated Protein Kinase by Metformin Improves Left Ventricular Function and Survival in Heart Failure. Circ Res (2009) 104(3):403–11. 10.1161/CIRCRESAHA.108.190918 19096023 PMC2709761

[45] Steen S Ingemansson R Eriksson L Pierre L Algotsson L Wierup P First Human Transplantation of a Nonacceptable Donor Lung after Reconditioning *Ex Vivo* . Ann Thorac Surg (2007) 83(6):2191–4. 10.1016/j.athoracsur.2007.01.033 17532422

